# Combination of chemotherapy and physical plasma elicits melanoma cell death via upregulation of SLC22A16

**DOI:** 10.1038/s41419-018-1221-6

**Published:** 2018-12-05

**Authors:** Sanjeev Kumar Sagwal, Gabriella Pasqual-Melo, Yana Bodnar, Rajesh Kumar Gandhirajan, Sander Bekeschus

**Affiliations:** ZIK plasmatis, Leibniz Institute for Plasma Science and Technology (INP Greifswald), Felix-Hausdorff-Str. 2, Greifswald, 17489 Germany

## Abstract

Malignant melanoma is an aggressive cancer that develops drug resistance leading to poor prognosis. Efficient delivery of chemotherapeutic drugs to the tumor tissue remains a major challenge in treatment regimens. Using murine (B16) and human (SK-MEL-28) melanoma cells, we investigated traditional cytotoxic agents in combination with cold physical plasma-derived oxidants. We report synergistic cytotoxicity of doxorubicin and epirubicin, and additive toxicity of oxaliplatin with plasma exposure in coefficient of drug interaction analysis. The combination treatment led to an increased DNA damage response (increased phosphorylation of ATM, γ-H2AX foci, and micronuclei formation). There was also an enhanced secretion of immunogenic cell death markers ATP and CXCL10 in cell culture supernatants following combination treatment. The observed synergistic effects in tumor cells was due to enhanced intracellular doxorubicin accumulation via upregulation of the organic cationic transporter SLC22A16 by plasma treatment. The doxorubicin uptake was reversed by pretreating cells with antioxidants or calcium influx inhibitor BTP2. Endoribonuclease-prepared siRNAs (esiRNA)-mediated knockdown of SLC22A16 inhibited the additive cytotoxic effect in tumor cells. SK-MEL 28 and THP-1 monocytes co-culture led to greater THP-1 cell migration and SK-MEL-28 cytotoxicity when compared with controls. Taken together, we propose pro-oxidant treatment modalities to sensitize chemoresistant melanoma cells towards subsequent chemotherapy, which may serve as therapeutic strategy in combination treatment in oncology.

## Introduction

The incidence of cutaneous melanoma has been steadily increasing in Europe and worldwide in the last three decades^1^. Treatment against melanoma includes conventional chemotherapy^2^, biochemotherapy (chemotherapy combined with interleukin-2 and interferon-α)^3^, small molecules against mutant BRAF^4^, and immune checkpoint inhibitors (anti-CTL4 and anti-PD1)^5^. Despite advances in treatment approaches, tumor heterogeneity confers varying degree of resistance and survival advantages limiting disease-free survival in patients^6^. Hence, there is the constant need in understanding tumor biology and optimize existing or develop novel combination therapeutic strategies.

Different classes of anti-neoplastic agents are employed as the frontline defense against cancers^7^. Most of these agents target or alter DNA synthesis and repair mechanisms leading to cell cycle arrest and death^8^. Doxorubicin (DOX) and epirubicin (EPI) are anthracyclines that intercalate DNA base pairs and inhibit topoisomerase II activity leading to DNA damage^9^. Oxaliplatin (OXA) is a platinum-based compound that reacts with DNA resulting in cell cycle-independent cell death. Vorinostat (VOR) is a histone deacetylase inhibitor that alters expression of tumor-suppressor and immunomodulatory genes, thereby showing clinical benefit^10,11^. Despite the effectiveness of these agents against multiple tumors, clinical trials using single-agent therapies have been not been satisfactory. Meta-analysis of systemic treatments in cutaneous melanoma reveals that combination therapy with anti-neoplastic agents, small molecules, and/or monoclonal antibodies improve overall survival of patients^12^. Importantly, oxidative stress seems key to control melanoma metastasis and progression^13–15^.

Cold physical plasma is a partially ionized gas that generates a multitude of reactive oxygen and nitrogen species (ROS/RNS)^16–18^. Cold plasma gained considerable interest due to its selective targeting of melanoma and multiple other cancer types in vitro and in vivo^19–27^. Oxidant overload presumably lead to mitochondria and endoplasmic reticulum dysfunction and subsequent apoptosis^28^. Recently, a clinical benefit in the palliation of 10 patients with advanced head and neck cancers has been provided using the accredited argon plasma jet kINPen^29^. In the current study, we asked what role cold plasma-derived oxidants play in combination with different classes of anti-neoplastic drugs. Using human and murine tumorigenic melanoma cell lines, we found that combination treatment DOX, EPI, or OXA and plasma significantly increased tumor cell killing in two-dimensional (2D) and three-dimensional (3D) cultures. The enhanced cell death was triggered by plasma-derived oxidants induced upregulation of the xenobiotic transporter SLC22A16 that led to increasing drug uptake and enhanced immunogenic cell death. Our findings suggest that cold plasma may serve as an additional tool in existing cancer therapy regimens to potentially improve clinical outcome.

## Materials and methods

### Cell culture and plasma treatment

B16F0, B16F10, SK-MEL 28, MDA-MD231, MCF10A, PC-3, and SW480 cells were subcultured in high glucose Dulbecco's minimum essential media (DMEM; Invitrogen) supplemented with 10% fetal calf serum (FCS). THP-1 cells were maintained in Roswell Park Memorial Institute 1640 (RPMI-1640; Invitrogen). In all, 1 × 10^4^ cells were seeded and incubated with indicated concentrations of DOX, EPI, OXA, or VOR (all Sigma) in RPMI-1640 with 2% FCS for 24 h in 96-well plates. Dimethylsulfoxide (Sigma) was used as vehicle control. The atmospheric pressure argon plasma jet kINPen (neoplas tools) served as reactive species-generating source and was operated at a frequency of 1 MHz with a feed gas flux of 3 l per minute of argon gas (99.9999% purity; Air Liquid). Argon gas only treated medium (with plasma off) served as control throughout all experiments.

### Metabolic activity and cell viability

To assess metabolic activity, 1 × 10^4^ cells were plated in 96-well culture plates (Nunc) in complete DMEM. Sixteen hours later, cells were challenged with indicated concentrations of DOX, EPI, OXA, or VOR in RPMI-1640 with 2% FCS for 24 h. Cells were then exposed to physical plasma (30 s) and further incubated for 6 h. Subsequently, wells were loaded with 100 µM of resazurin (Alfa Aesar) that is transformed to fluorescent resorufin by metabolically active cells. The plate was incubated for 2 h at 37 °C, and fluorescence was measured in multimode plate reader (Tecan) at λ_ex_ 535 nm and λ_em_ 590 nm. Metabolic activity is shown as percent of untreated control. Dose-response curves and IC_50_ values were generated by nonlinear regression analysis. The nature of drug interaction was analyzed by the coefficient of drug interaction (CDI), which was calculated as CDI = AB/(A × B). According to the absorbance of each group, AB is the ratio of the two-drug combination group to the control group, and A or B is the ratio of the single-agent group to the control group. Thus, a CDI value of < 1, = 1, or > 1 indicates that the drugs are synergistic, additive, or antagonistic, respectively^30,31^. To determine toxicity, cells were loaded with sytox green (1 µM; Thermo Fisher), for 30 min at 37 °C. Cells were imaged with a × 20 objective using a live cell high-throughput imaging system (Operetta CLS; PerkinElmer) and quantified using dedicated image analysis software (Harmony 4.6; PerkinElmer).

### Small interfering RNA-mediated knockdown of SLC22A16

SK-MEL-28 cells (1 × 10^4^) were seeded in 96-well plates. esiRNA against human SLC22A16 (Sigma-Aldrich) or non-targeting control esiRNA (Luc) was transfected using X-tremeGENE siRNA reagent (Sigma-Aldrich) according to manufacturer’s recommendation. Whole-cell lysates were made after 48 h to confirm the knockdown efficiency of SLC22A16 by immunoblotting.

### Immunoblotting

Cells were harvested in ice-cold phosphate-buffered saline (PBS). Harvested cells were lysed in RIPA buffer (Cell signaling) supplemented with complete protease and phosphatase inhibitors (PIM complete; Roche) for 20 min on ice. After centrifugation at 15,000 *g* for 15 min at 4 °C, total protein in whole-cell extracts was quantified using Rotiquant (Carl Roth). In all, 40 µg of protein was resolved by sodium dodecyl sulfate–polyacrylamide gel electrophoresis (Invitrogen) and blotted on polyvinylidene fluoride membranes (Invitrogen). The membranes were probed with anti-OCT6 or anti-β actin (Santa Cruz) primary antibodies followed by secondary horse-radish peroxidase-coupled antibodies (Rockland Immunochemicals), and signals were acquired in a chemiluminescence detection system (Applied Biosystems) in a linear dynamic range.

### Micronucleus assay and immunofluorescence

In all, 1 × 10^4^ cells were plated in 96-well culture plates (Nunc) in complete DMEM. Sixteen hours later, cells were challenged with indicated drugs for 24 h. Cells were then exposed to plasma (30 s) and further incubated for 6 h. Cells were then fixed in 4 % paraformaldehyde (Sigma) for 30 min. Cells were washed in PBS and counterstained with Hoechst 33258 and cell trace yellow (Thermo Fisher), and imaged with a × 40 water immersion objective using a live cell high throughput imaging system (Operetta CLS; PerkinElmer). Micronuclei were detected and quantified using dedicated image analysis software (Harmony 4.6; PerkinElmer).

For immunofluorescence, cells were blocked in 5% normal serum/0.3% Triton X-100 in PBS for 1 h. Cells were then incubated with anti phospho-ATM and anti phospho-H2AX (γ-H2AX) antibodies (Cell Signaling, 1:1000) diluted in 1% bovine serum albumin/0.3% Triton X-100 in PBS overnight at 4 °C. Cells were washed three times with PBS, followed by incubation with diluted secondary fluorescent antibodies (Alexa Flour 647; 1:5000) for 1 h. After final washing, cells were counterstained with PBS containing Hoechst 33258 (Thermo Fisher) and imaged using a × 40 water objective in high-throughput imaging system (Operetta CLS; PerkinElmer). Image analysis and quantification was performed by dedicated imaging software (Harmony 4.6; PerkinElmer)

### ATP assay

Cells (1 × 10^4^) were seeded in 96-well plates overnight. Cells were then treated with indicated conditions as described earlier in triplicates, and ATP levels were quantified in fresh supernatants using a luminescence-based ATP determination kit (Enzo Life Sciences) according to manufactures’ instructions.

### ELISA

Cell culture supernatants were used to perform enzyme-linked immunosorbent assays (ELISAs) for human CXCL10 and HMGB1 (Thermo Fisher) according to manufacturer’s instructions.

### Spheroid assay

SK-MEL-28 cells (6 × 10^3^) were incubated in 96-well, round-bottom, ultra-low affinity plates (PerkinElmer). Seventy-two hours later, spheroids had formed and were challenged with treatment regimens as described above. Twenty-four hours later, spheroids were loaded with sytox orange (5 µM; Thermo Fisher) and Hoechst (10 µM; Thermo Fisher) for 1 h at 37 °C. Spheroids were imaged with a × 5 objective using 50 stacks per well imaged with a live cell high-throughput imaging system (Operetta CLS; PerkinElmer) and quantified using dedicated imaging software (Harmony 4.6; PerkinElmer).

### Transwell migration assay

Following combination treatment, cell culture supernatants (500 µl) were used for transwell migration assay for 72 h in Boyden’s chamber (Nunc) according to manufacturer’s instructions and using THP-1 cells. Migrated cells were stained with DRAQ5 (1 µM; Thermo Fisher) and images were acquired by high-throughput confocal imaging (Operatta CLS; PerkinElmer) with a × 5 objective. Cells were then trypsinized, and total cell counting was performed using flow cytometry (Attune; Applied biosystems).

### Co-culture

In all, 6 × 10^3^ SK-MEL-28 cells were seeded in 96-well plates overnight. Cells were treated with 0.1 µM of different chemotherapeutic drugs for 24 h, followed exposure to cold physical plasma (30 s). Six hours later, 5 × 10^3^ THP-1 monocytes, pre-loaded with cell trace far red (5 µM; Thermo Fisher) and sytox green (1 µM; Thermo Fisher) was added to each well. Time-lapse imaging was commenced with intervals of 30 min for 72 h using a high-throughput imaging system (Operetta CLS; PerkinElmer). Quantification of viability and mean square displacement was performed by dedicated imaging software (Harmony 4.6; PerkinElmer) based on cell staining and size (µm).

### Intracellular accumulation of DOX

To measure DOX uptake, 1 × 10^4^ cells were seeded in 96-well plates overnight. The next day, the cells were incubated with free DOX (5 µM) for 6 h at 37 °C. Cells were washed twice with PBS followed by lysis in RIPA buffer. Total cell lysates was collected by centrifugation at 15,000 *g* and 4 °C for 15 min, and fluorescence intensity of accumulated DOX was measured in multimode plate reader (Tecan) at λ_ex_ 450 nm and λ_em_ 590 nm. The amount cellular protein present in lysate was also calculated using the bicinchoninic acid protein assay kit (Carl Roth) following the protocol of the manufacturer. Finally, the amount of DOX taken up by the specific cell type was determined by normalizing fluorescence intensity with total protein content. For intracellular localization studies, DOX was measured (λ_ex_ 450 nm and λ_em_ 610 ± 40 nm) with a live cell high-throughput imaging system (Operetta CLS; PerkinElmer) and quantified using dedicated imaging software (Harmony 4.6; PerkinElmer).

### Quantitative real-time PCR

Following esiRNA knockdown, total mRNA was isolated using RNA isolation kit (BioSell GmbH). In all, 1 µg of mRNA was converted to complementary DNA (cDNA) using High-Capacity cDNA Reverse Transcription Kit (Thermo Scientific). Predesigned KiCqStart™ SYBR green primers for human beta actin, SLC22A16, SLC22A2, and SLC22A3 were obtained from Sigma-Aldrich. Quantitative PCR assays were carried out using Power SYBR™ Green PCR Master Mix (Thermo Fisher) according to manufacturer’s instructions.

### Statistical analysis

Graphing and statistical analysis was performed using prism 7.05 (GraphPad software). The difference between groups was analyzed using a Student’s *t*-test when comparing only two groups or one-way analysis of variance when comparing more than two groups with the Tukey’s multiple comparison test. The *p-*values < 0.05 were considered statistically significant.

## Results

### Plasma treatment exerts additive toxic effects in drug-sensitized melanoma cells

**Fig. 1:**
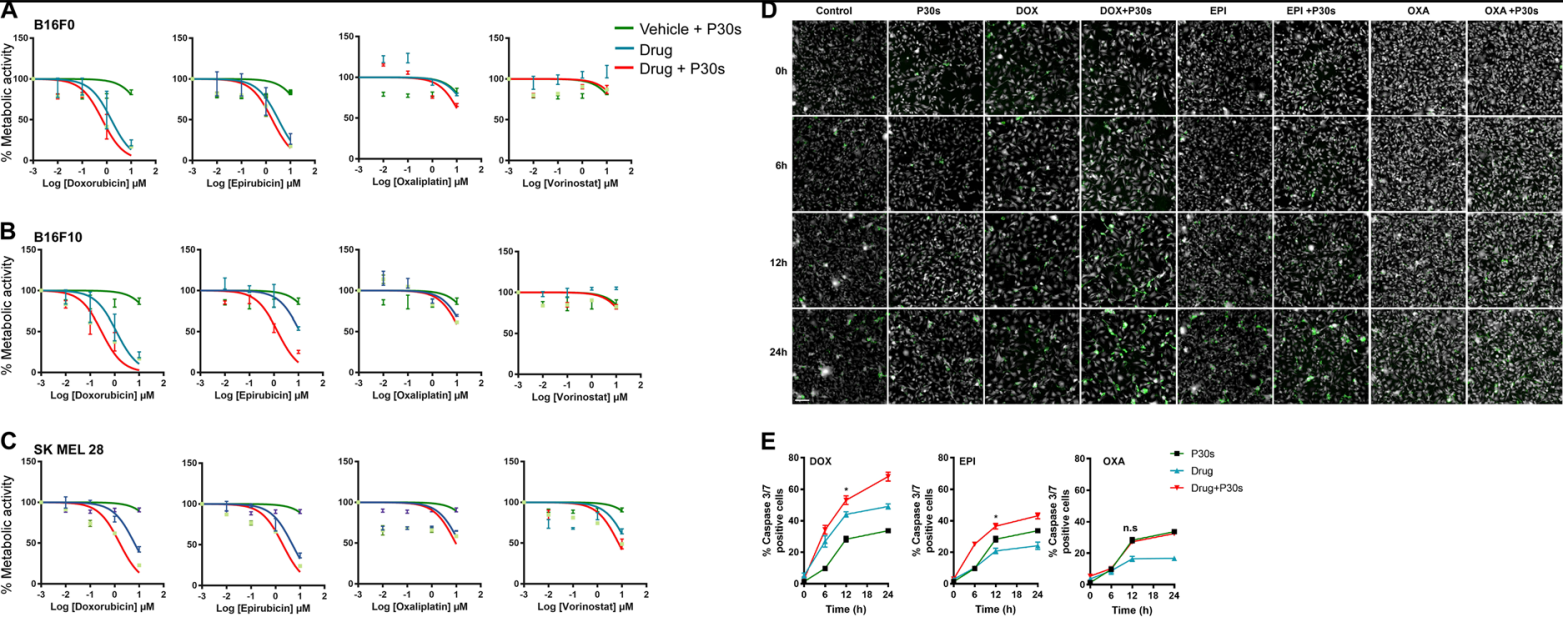
Plasma treatment exerts synergistic toxic effects in drug-sensitized melanoma cells. **a**–**c** Dose-response curves of metabolic activity of three melanoma cell lines upon 24-h pretreatment with chemotherapeutics (0.01, 0.1, 1.0, and 10 µM) either followed by exposure to cold physical plasma or not. **d, e** Representative time-lapse images and quantification of caspase 3/7 activation in B16F10 cells pretreated with 0.1 µM of chemotherapeutics followed by plasma treatment. Results are shown as mean ± SEM. Significance determined by one-way ANOVA with Tukey post-test for multiple comparisons derived from three independent experiments. Scale bar: 100 µm

**Fig. 2:**
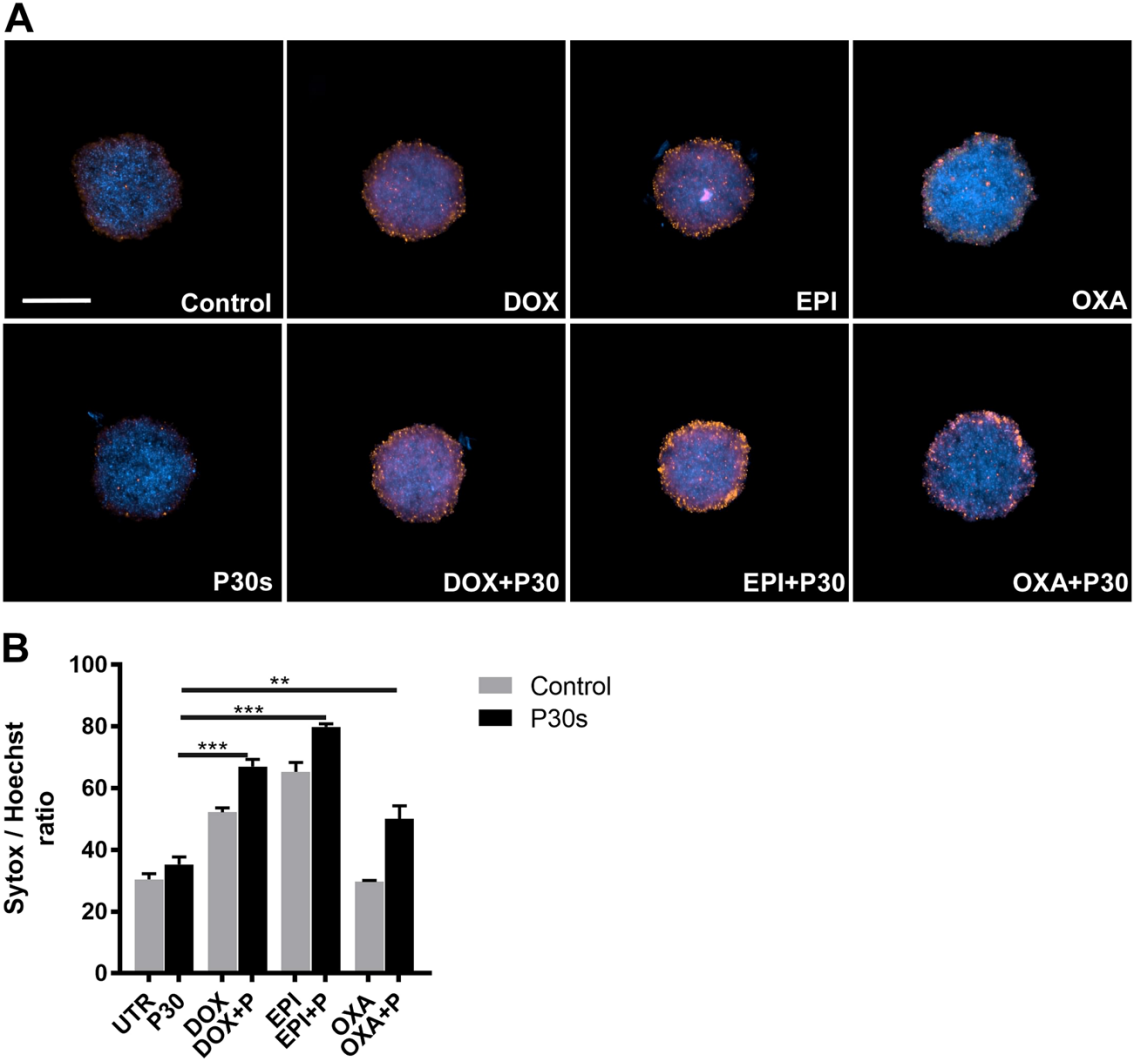
Plasma treatment increased toxicity in tumor spheroids pretreated with cytotoxic agents. **a** SK-MEL 28 tumor spheroids were treated with 1 µM of indicated drugs for 24 h, followed by exposure to cold physical plasma and further incubation for 24 h before staining with Hoechst and sytox orange. **b** Quantification of tumor cell toxicity. Results are shown as mean ± SEM. **p < 0.01; ***p < 0.001. Significance determined by one-way ANOVA with Tukey post-test for multiple comparisons. Scale bar: 250 µm

To test the toxicity of combination treatment using cold plasma-derived oxidants and anti-neoplastic agents, melanoma cells (B16F0, B16F10, and SK-MEL-28) were pretreated with increasing concentrations (0.01, 0.1, 1.0, and 10 µM) of DOX, EPI, VOR, or OXA. A dose-dependent reduction in metabolic activity was observed in all cell lines and drugs (Figs. 1a, c). For plasma treatment, a short exposure time (30 s) with low inherent toxicity was chosen. Upon combination of drug and plasma treatment, however, IC_50_ values were reduced compared with drug treatment alone in SK-MEL-28: DOX (6.40 ± 0.64 vs 1.62 ± 0.09 µM), EPI (4.89 ± 0.43 vs 1.99 ± 0.64 µM), OXA (13.74 ± 0.24 vs 9.52 ± 0.19 µM), and VOR (17.23 ± 1.62 vs 15.81 ± 1.87 µM). Similar results were obtained in B16F0 and B16F10 cell lines except VOR did not show cytotoxicity in murine cells (Table 1). CDI analysis reveal synergistic effect for DOX and EPI and additive effect for OXA in the combination treatment (Table 2). We further enumerated caspase 3/7 activation in B16F10 cells by live time-lapse microscopy following cold plasma treatment. There was a significant induction of caspase 3/7 activity upon combination therapy (DOX and EPI) when compared with monotherapy (Figs. 1d, e). However, OXA failed to induce caspase 3/7 activation at the concentration tested consistent with metabolic activity assays. We further confirmed the cytotoxicity by sytox green staining and live microscopy at 6 h following treatment (Fig. S1). There was a significant increase in cell death when compared with singular treatment modalities in B16F10 and SK-Mel 28 cells. To reinforce the data obtained in 2D culture, 3D tumor spheroid were generated using SK-Mel 28. Spheroids were pretreated with of DOX, EPI, or OXA for 24 h (1 µM) followed exposure to physical plasma. One day later, live cells (Hoechst) and dead cells (sytox green) nuclei were labeled, and z stack images were acquired. Representative maximum intensity projections (Fig. 2a) and fluorescence quantification (Fig. 2b) revealed enhanced toxicity in spheroids upon combination treatment. These data demonstrate that plasma-derived oxidants lead to synergistic or additive toxicity in melanoma cells grown sensitized with DOX, EPI, or OXA.

**Table 1:** Tabulation of IC_50_ values following combination treatment with indicated drugs

Cell line	EPI (µM ± SEM)	EPI + P30s (µM ± SEM)	DOX (µM ± SEM)	DOX + P30s (µM ± SEM)	OXA (µM ± SEM)	OXA + P30s (µM ± SEM)	VOR (µM ± SEM)	VOR + P30s (µM ± SEM)
SKM-MEL 28	4.89 ± 0.43	1.99 ± 0.19	6.40 ± 0.64	1.62 ± 0.09	13.74 ± 0.24	9.524 ± 0.19	17.23 ± 1.62	15.81 ± 1.87
B16F10	11.62 ± 1.63	1.18 ± 0.21	1.18 ± 0.75	0.29 ± 0.45	21.04 ± 1.0	14.45 ± 0.51	NA	NA
B16F0	4.42 ± 1.23	2.10 ± 0.69	1.54 ± 0.71	0.66 ± 0.49	37.04 ± 1.34	16.35 ± 1.42	NA	NA

**Table 2:** Coefficient of drug interaction (CDI) of chemotherapeutic drugs and cold plasma in melanoma cells

	P30s						
CDI	Cell line	EPI (1 µM)	EPI (10 µM)	DOX (1 µM)	DOX (10 µM)	OXA (1 µM)	OXA (10 µM)
	B16F0	0.56	0.91	0.82	0.96	1.00	1.01
	B16F10	0.77	0.84	0.76	0.85	1.09	1.05
	SK-Mel 28	0.86	0.91	0.74	0.93	1.02	1.06

### Physical plasma–drug combination treatment was affected via the DNA damage response

**Fig. 3:**
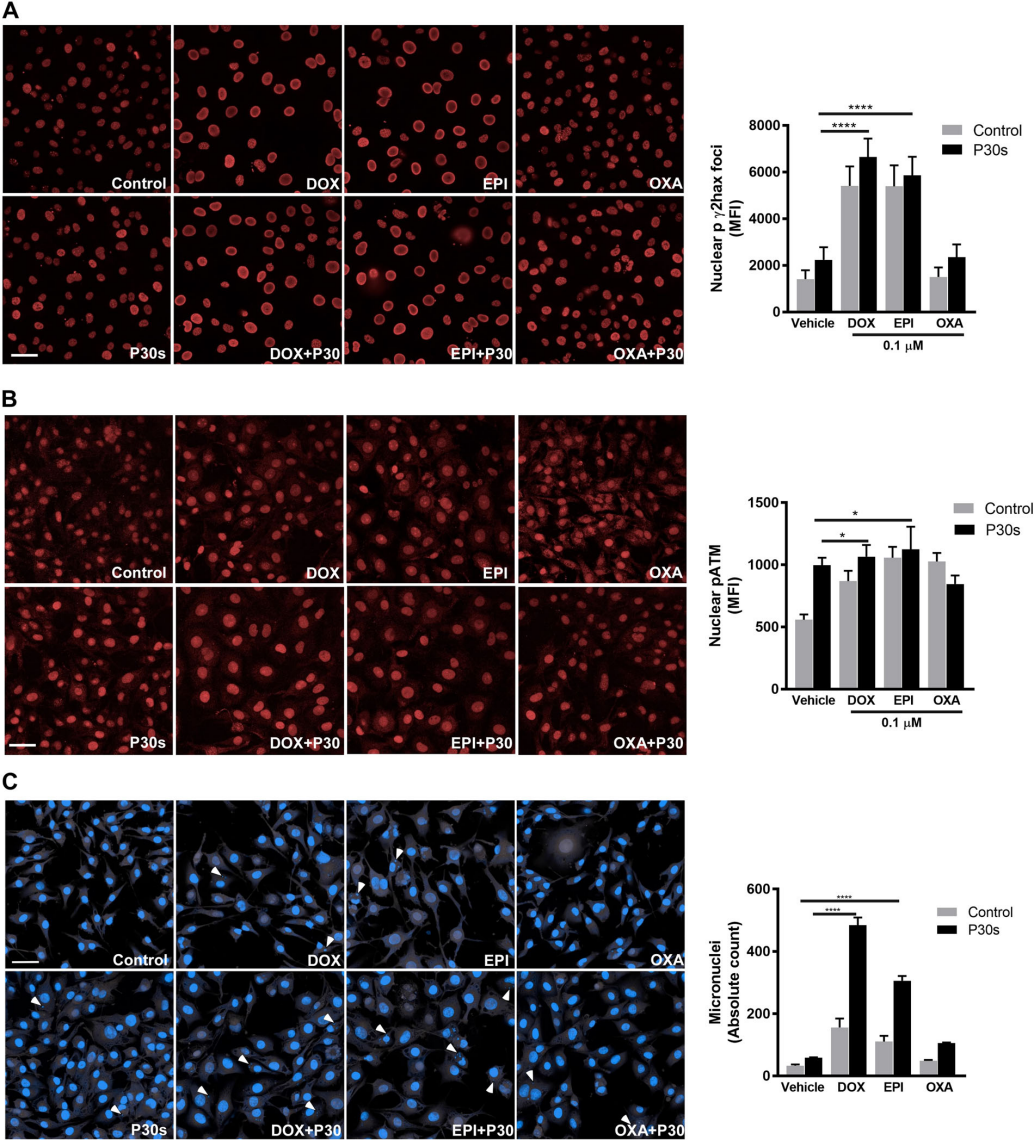
Chemotherapeutics and plasma treatment increased DNA damage response in melanoma cells. SK-MEL 28 cells were exposed to combination treatment as indicated. Cells were fixed and stained with anti γ-H2AX (**a**) or anti pATM (**b**) antibodies for immunofluorescence, and nuclear fluorescence was quantified. **c** Representative images and quantification of micronuclei in SK-MEL 28 cells following combination treatment. Data are represented as mean ± SEM derived from three independent experiments. **p < 0.01; ****p < 0.0001. Significance determined by one-way ANOVA with Tukey post-test for multiple comparisons. Scale bar: 100 µm

The chemotherapeutic agents used in our study induce cytotoxicity by interfering with DNA synthesis resulting in double-strand breaks (DSBs) leading to checkpoint arrest and apoptosis. This induces the DNA damage response mediated by phosphorylation of ATM and γ-H2AX. Formation of pATM and γ-H2AX foci in the nuclei is used as a marker for DSBs in cells. Since an enhanced toxicity following combination treatment was observed, the hypothesized was that a concomitant increase in DNA damage response may account for the results observed. Hence, phosphorylation status of ATM and formation of γ-H2AX foci in SK-MEL 28 cells was observed following combination treatment. A significant increase in nuclear pATM and γ-H2AX foci was identified in cells treated with drugs alone. However, these levels were significantly amplified with combination treatment (Figs. 3a, b) indicating cold plasma aggravating DNA damage particularly with anthracyclines (DOX or EPI). A considerable increase of these markers was also seen in cells treated with cold plasma alone. Finally, we confirmed the DNA damage response by assessing micronucleus formation following combination treatment. Interestingly, exposure to plasma alone did not induce significant micronucleus formation. However, cytotoxic agents induced a significant increase in micronucleus formation at 6 h post combination treatment (Fig. 3c).

### Plasma treatment increase intracellular DOX in SK-MEL 28 melanoma cells via upregulation of SLC22A16

**Fig. 4:**
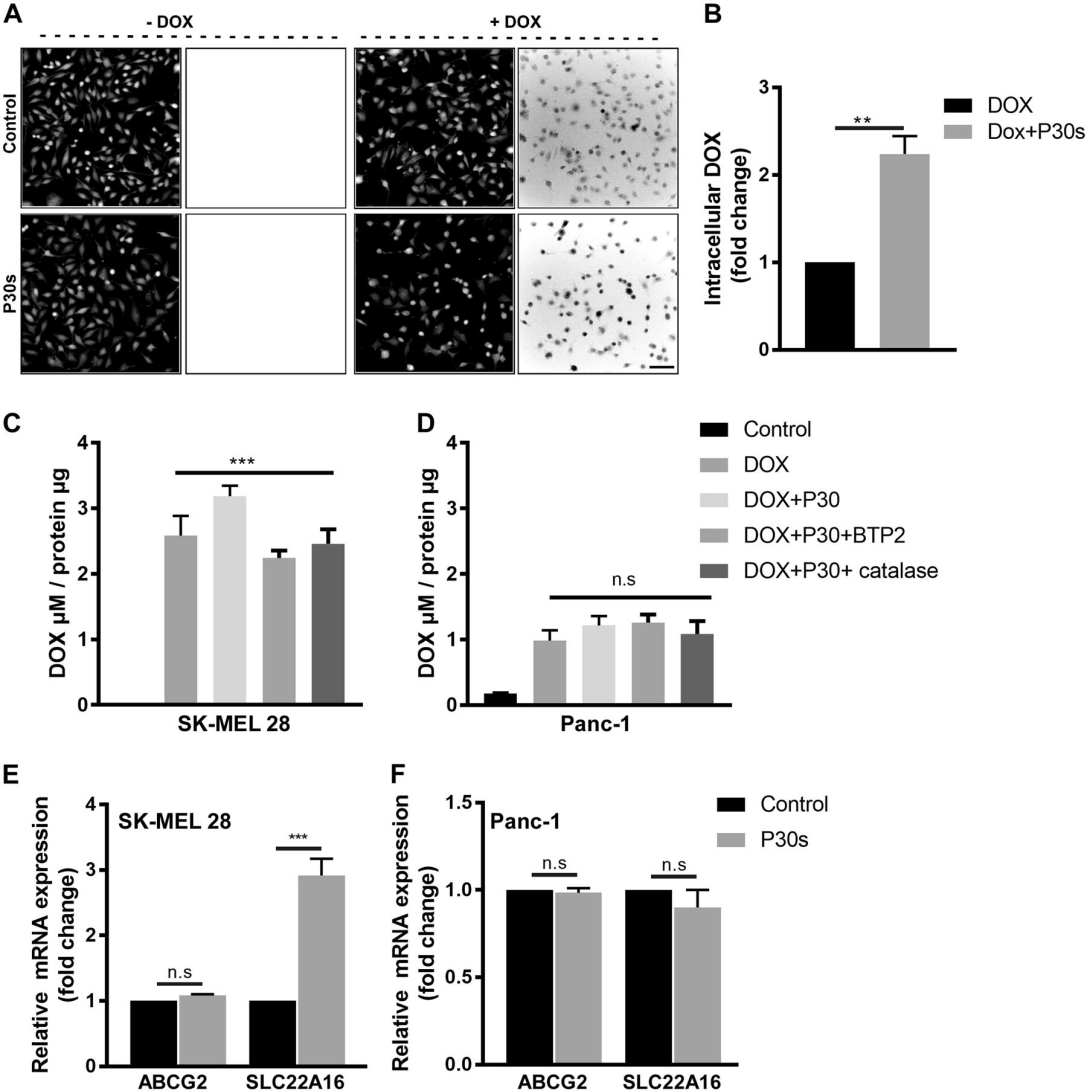
Plasma treatment increased DOX uptake by inducing SLC22A16 expression. **a** Representative images and **b** quantification of DOX (5 µM) autofluorescence in SK-MEL 28 cells pretreated for 5 h followed by cold plasma exposure. **c**, **d** Quantification of intracellular DOX levels in SK-MEL 28 and Panc 1 cells, respectively. **e**, **f** Relative fold change in mRNA expression levels of ABCG2 and SLC22A16 in SK-MEL 28 and PANC 1 cells at 6 h following plasma exposure. Data are mean + SEM from three independent experiments. Significance determined by unpaired Student’s *t-*test with Welch’s correction (**b**, **e,** **f**) or one-way ANOVA with Tukey post-test for multiple comparisons (**c**, **d**). **p < 0.01; ***p < 0.001; n.s. not significant. Scale bar: 100 µm

An increased DNA damage response and associated tumor cell toxicity was observed, leading us to investigate the intracellular concentration of DOX following plasma treatment. Autofluorescence quantification of DOX with cells treated with cold plasma showed a twofold increase in intracellular localization in SK-MEL 28 cells (Figs. 4a, b). To validate this observation, we quantified the intracellular DOX by normalizing DOX concentration with total protein content in whole-cell lysates as previously described^32^. The results specify that there was increased uptake of DOX 6 h post combination treatment in SK-MEL 28 cells. This enhanced uptake was reversed by incubating the cells with exogenous catalase or calcium release-activated channel inhibitor BTP2 indicating plasma-derived oxidants (specifically hydrogen peroxide) and Ca^2+^ influx playing a major role in either retention or uptake of DOX into the SK-MEL 28 cells (Fig. 4c). However, this observation was not observed in the pancreatic cancer cell line Panc-1 (Fig. 4d). To understand the mechanism involved, we quantified the mRNA expression of the organic cation importer SLC22A16 and the xenobiotic exporter ABCG2 in cells treated with plasma for 6 h. Interestingly, plasma treatment preferentially upregulated the expression of SLC22A16 twofold (Fig. 4e) but levels of ABCG2 remained unaltered (Fig. 4f). SLC22A16 gene expression following exposure to plasma was also investigated in other tumor cell lines PC-3, MDA-MD231, MCF10A, and SW480 (Fig S2) with the first two showing an upregulation while the latter two did not.

### Upregulation of SLC22A16 is essential for toxicity following drugs and plasma treatment

**Fig. 5:**
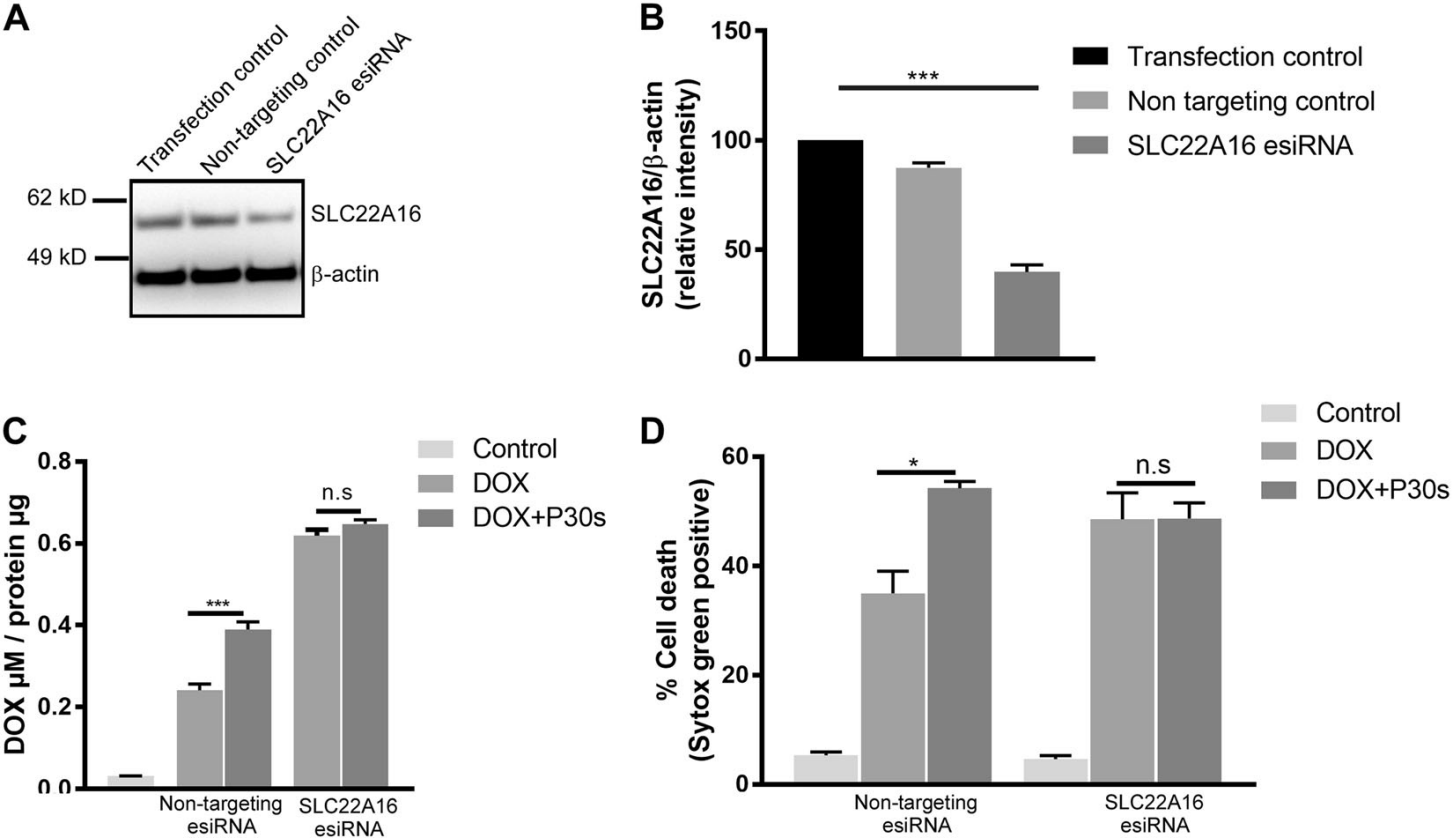
Cold physical plasma induced expression of SLC22A16 is essential for synergistic cytotoxic effects in combination treatment. **a** Immunoblot and **b** densitometric analysis of SK-Mel-28 cell lysates against SLC22A16 following transfection with esiRNA with appropriate controls at 48 h. **c** Quantification of intracellular DOX levels in SK-Mel 28 following knockdown of SLC22A16 and combination treatment. **d** Quantification of cytotoxicity following knockdown of SLC22A16 and combination treatment in SK-MEL 28 cells. Data are mean + SEM from three independent experiments. Significance determined by one-way ANOVA with Tukey post-test for multiple comparisons (**b**) or unpaired Student’s *t*-test with Welch’s correction (**c**, **d**). *p < 0.05; ***p < 0.001; n.s. not significant

To validate that cold plasma induced expression of SLC22A16 was essential for increased DOX cytotoxicity, esiRNA knockdown experiments against SLC22A16 were performed. Knockdown of SLC22A16 led to >50% reduction in its protein levels after 48 h (Figs. 5a, b). Melanoma cells were then treated with combination treatment, and intracellular DOX levels and tumor cell toxicity was performed as previously described. Knockdown of SLC22A16 increased basal levels of DOX in SK-MEL 28 cells, however, there was no further DOX uptake upon plasma exposure (Fig. 5c). Similarly, there was no additive cytotoxic effect seen in combination treatment in knockdown cells (Fig. 5d). We speculate this is due to a dosage compensatory effect by expression of other transporters of SLC family. We show a significant increase in expression of SLC22A2 and SLC22A3 in SKM-MEL 28 cells following SLC22A16 knockdown (Fig S3). These results suggest that cold plasma induced expression of SLC22A16 is critical for enhanced toxicity observed drug-treated melanoma cells.

### Plasma and drug treatment enhanced melanoma cell release of ATP and CXCL10 with concomitantly enhanced chemotaxis of THP-1 monocytes that augmented tumor cell killing

**Fig. 6:**
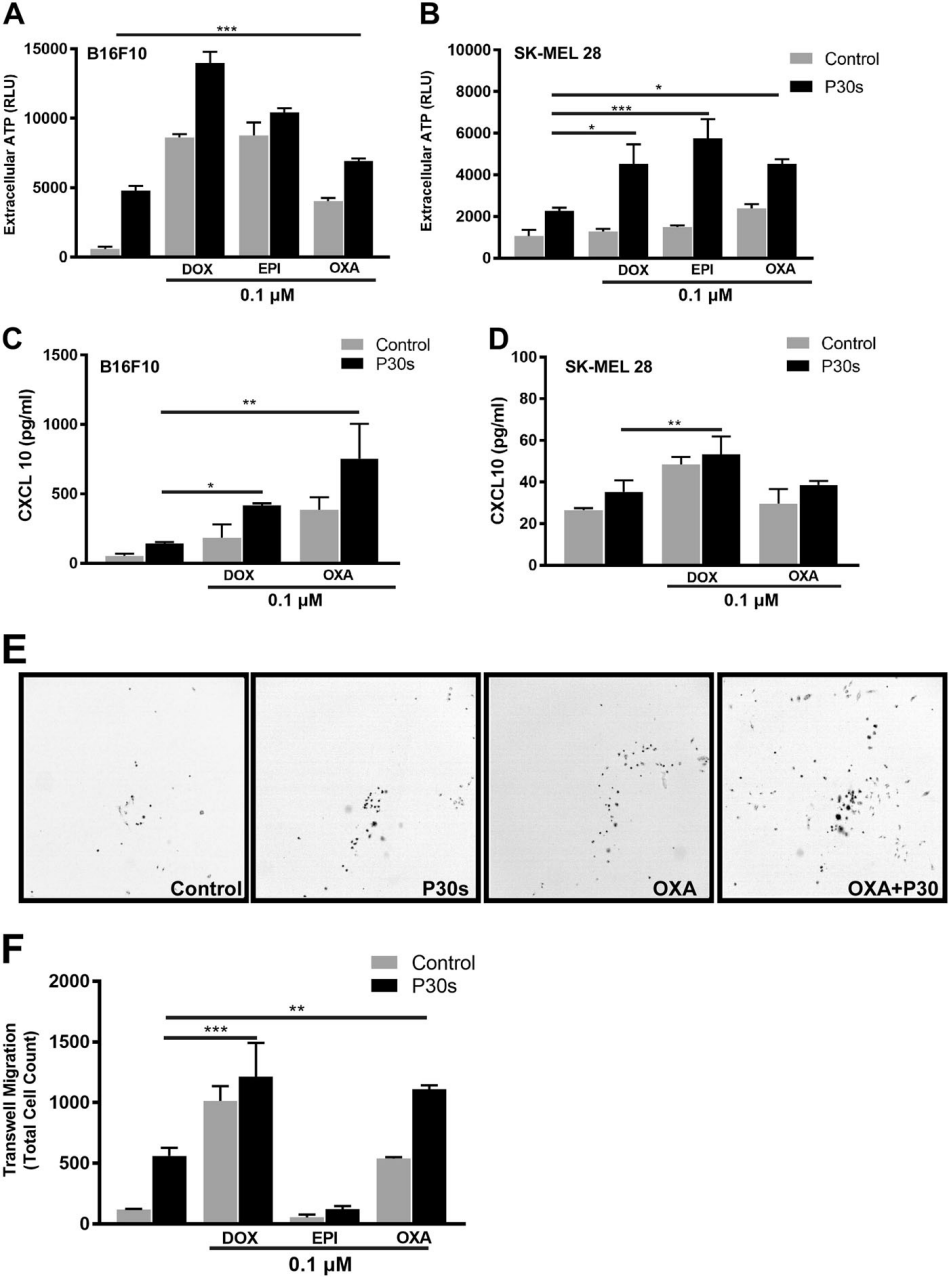
Combination treatment led to secretion of ATP and CXCL10 concomitant with enhanced chemotaxis of THP-1 monocytes. **a**, **b** ATP and CXCL10 levels in cell culture supernatants of B16F10 and SK-MEL 28 cells following combination treatment. **c** Cell culture supernatants from SK-MEL 28 cells were incubated with THP-1 monocytes in Boyden’s chamber for 72 h. **c** Representative images of migrated THP-1 monocytes from supernatants derived from OXA. **d** Quantification of total migrated cells after 72 h from wells containing respective supernatants. Data are mean + SEM from three independent experiments. Significance determined by one-way ANOVA with Tukey post-test for multiple comparisons (**a**–**f**). *p < 0.05; **p < 0.01; ***p < 0.001; n.s = not significant

**Fig. 7:**
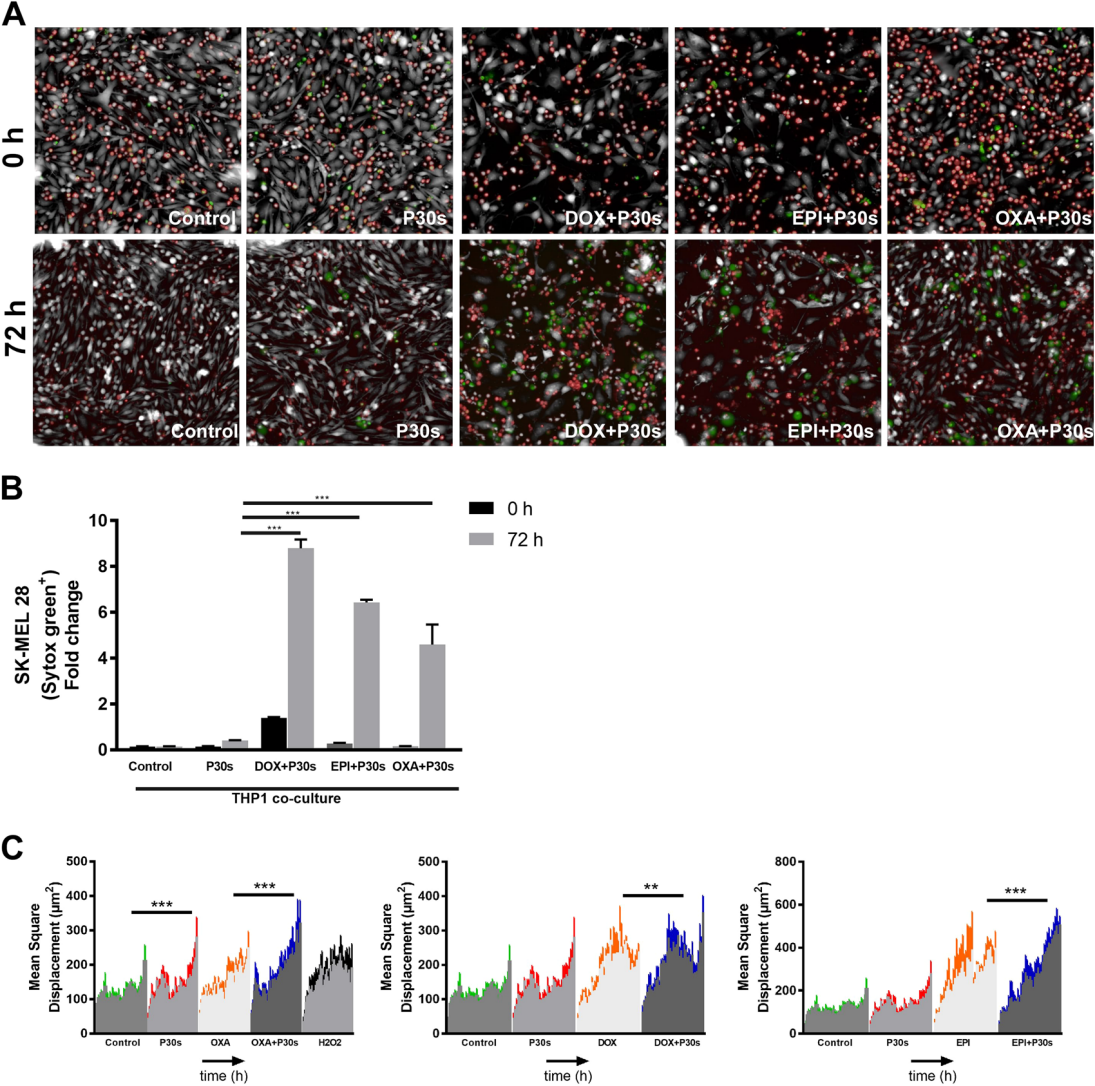
THP-1 monocytes potentiated killing of melanoma cells upon combination treatment. **a** Representative images from time-lapse experiments show SK-MEL 28 cells (gray), THP-1 monocytes (red), and dead cells (green) at 0 and 72 h post combination treatment, respectively. **b** Quantification of dead cells/THP-1 monocyte ratio from (**a**) at 0 and 72 h. **c** Mean square displacement of THP-1 monocytes by time-lapse imaging of co-cultures over a period of 40 h. Data are mean + SEM of from three independent experiments. Significance determined by one-way ANOVA with Tukey post-test for multiple comparisons (**b**) or unpaired Student’s *t*-test with Welch’s correction (**c**). **p < 0.01; ***p < 0.001

Plasma treatment of melanoma cells led to a fourfold elevation of extracellular ATP, and combination treatment with chemotherapeutic agents enhanced this effect in B16F10 (Fig. 6a) and SK-MEL 28 (Fig. 6b) cells. Similarly, there was enhanced secretion of chemoattractant CXCL10 in the combination setting in B16F10 (Fig. 6c) and SK-MEL 28 (Fig. 6d) cells. Concomitant with the release of ATP and CXCL10, increased migratory activity of THP-1 monocytes to tumor cell culture supernatants was observed qualitatively (Fig. 6e) and quantitatively (Fig. 6f), and especially in combination treatment regimens. To assess a possible contribution of THP-1 monocytes in tumor cell killing, fluorescently labeled THP-1 cells were co-cultured up to 72 h with SK-MEL 28 cells 6 h after pre-exposition to mono and combination treatment regimens. Live cell imaging revealed a significant increase in sytox green positive (dead) cells in the fraction of cells that were segmented as melanoma (phase contrast-positive, fluorescent label-negative) cells (Fig. 7a; Supplemental videos 1, 2, and 3). Tumor cell killing in presence of THP-1 cells was especially prominent in combination treatment regimens (Fig. 7b). Furthermore, the mean square displacement of THP-1 monocytes was calculated over period of 40 h. The results indicate a significant increase in motility of THP-1 cells in mono treatment regimens, which was significantly increased upon combination with OXA, DOX, or EPI, respectively (Fig. 7c).

## Discussion

Our data highlight the cytotoxic effects of cold plasma in combination with chemotherapeutic agents achieved at sub-micromolar drug concentrations. Initial experiments were performed to compare the sequence of combination therapy in tumor cells. Preliminary results indicated that pretreatment of the drug for 24 h followed plasma exposure had a significant decrease in metabolic activity when compared with co-treatment with drug and plasma (data not shown). Furthermore, we chose to perform the in vitro experiments with 10-fold less concentration than the IC_50_ values (DOX and EPI) (Table 1) to emphasize the desired effects can be achieved at sub-micromolar drug concentrations and within a safe exposure level of plasma-derived oxidants. Importantly, we demonstrate anthracyclines show a robust synergistic immunogenic and cytotoxic response when compared with platinum-based or histone deacetylase compounds in 2D and 3D culture. The efficacy bias towards anthracyclines was due to plasma induced expression of SLC22A16, which was previously described as DOX importer^33^.

ROS and RNS are known to evoke transient Ca^2+^ signaling leading to receptor-mediated signaling, proliferation, and/or apoptosis^34,35^. We observed an upregulation of the organic cation transporter SLC22A16 following exposure with cold plasma-derived oxidants in a subset of cancer cell lines, which was inhibited by antioxidant catalase and Ca^2+^ channel inhibitor BTP2. Expression of SLC22A16 play a critical role in drug uptake in tumors and several studies had warranted strategies to upregulate it for better clinical outcome^33,36^. Furthermore, SLC22A1 has been implicated for the uptake of several platinum-based drugs^37^, we speculate that lack of synergism with OXA is due to absence of plasma induced expression of SLC22A1 in melanoma cells (data not shown).

Increased DOX content in SLC22A16 knockdown cells could be attributed to upregulation of other transporters of the SLC family as previously seen in SCL22A1 knockout models^38–40^. We demonstrate similar findings in vitro wherein knockdown of SLC22A16 in melanoma cells led to increased expression of SLC22A1 and SLC22A3. Although, a previous study implicated SLC22A1 as an importer of anthracyclines^41^ our observations indicate plasma induced SLC22A16 expression but not SLC22A1 in melanoma cells. This could be cell-specific response and further studies are necessary to delineate the mechanisms involved. To strengthen our observations, mRNA levels of multiple drug resistance gene ABCG2 (ATP-binding cassette sub-family G member 2 with prime importance in, e.g., breast cancer^42^) remained unaltered in all cell lines tested.

Combination treatment led to decrease in IC_50_ values of all the drugs tested except histone histone deacetylase (HDAC) inhibitor VOR. In melanoma patients, the drug alone is not able to confer protective effects^43^. Previous studies indicate that HDAC inhibitors sensitize melanoma cells to immunotherapy^44^ possibly due to its endoplasmic reticulum stress and autophagy provoking activity^45^, two processes crucial in eliciting immunogenic cancer cell death^46^. However, we saw limited effect in monotherapy or in combination with plasma. We speculate the lack of substantial effect by VOR is due to its ability to induce expression of ABCG2 and activate drug efflux pathway^47^. Of note, one study found synergistic toxicity of VOR in combination with antioxidants^48^ as opposed to the pro-oxidant treatment regimen in our study.

Damage-associated molecular patterns (DAMPs) are biomolecules released by dying cells, which can attract and prime phagocytose to take and present tumor antigen to effector T cells^49^. DOX, EPI, and OXA were previously described to induce the release of DAMPs in multiple tumor cells^50–54^. Extracellular ATP is a well-known “find me” signal that is released from apoptotic cells. ATP binds to purinergic receptors in monocytes triggering chemoattraction^55^. CXCL10 plays an important role in recruitment and activation of monocytes by binding to G protein-coupled receptor CXCR3^56^. Interestingly, CXCL10 is also an attractor for lymphocytes^57^ and indispensable for antitumor immunity^58^. Our findings reveal an increase of secreted CXCL10 and ATP into the extracellular environment following combination treatment in melanoma cells. Besides, additive tumor toxicity was several fold increased upon THP-1 co-culture. Myeloid-dependent tumor cell killing was observed for decades^59^. It was recently established that not only T-cell mediated immunity but also tumor phagocytosis by monocyte/macrophages is targeted by checkpoint blockage^60^. THP-1 monocytes were observed to inactivate and phagocytose breast cancer cells in vitro^61^. Monocyte/macrophage-dependent killing mediated via, e.g., release of tumor necrosis factor-α (TNFα) or nitric oxide, and is enhanced by their activation with IFNγ^62^. Interestingly, a TNFα-dependent killing was suggested previously as effector mechanisms of raw264.7 macrophages potentiating plasma-mediated killing of cancer cells in vitro^63^.

The redox chemistry leading to plasma-mediated anticancer effects is only partially explored. The plasma gas phase and its spatio-temporal resolution reactive species efflux emitted by the jet utilized in this work is extensively studied^64–67^. Yet, reactive species chemistry in liquids and on cell membranes is far less understood. A large number of different species can be measured in plasma-treated liquid^68^, including hydrogen peroxide, superoxide, nitrite, singlet oxygen, and peroxynitrite^69–71^. Our results with catalase suggest H_2_O_2_ as a primary oxidant mediating cell death. However, plasma-derived H_2_O_2_ can act in concert and synergistically with other plasma-derived species such as superoxide^72^ and nitrogen dioxide^73^, as well as oxidases on cell membranes^74,75^. This is well illustrated in a study using the plasma jet and neutrophils, where catalase was able to inhibit plasma-mediated effects without H_2_O_2_ being able to replicate it^76^. In another study, atomic oxygen and its downstream products but not H_2_O_2_ were central for plasma-mediated killing^77^. Further studies are necessary to understand the delicate interplay between plasma-derived oxidants, drug transporter regulation, tumor cell killing, and immunological determinants to find and characterize novel tumor cell death avenues for oncotherapy.

## Electronic supplementary material

Figure S1

Figure S2

Figure S3

Supplementary video 1

Supplementary video 2

Supplementary video 3

Supplementary figure legends
