# Supplementary figures and images for: Combination of chemotherapy and physical plasma elicits melanoma cell death via upregulation of SLC22A16
Source: Cell Death Dis. 2018 Dec 5;9(12):1179. doi: 10.1038/s41419-018-1221-6 (PMC6281583; doi:10.1038/s41419-018-1221-6)

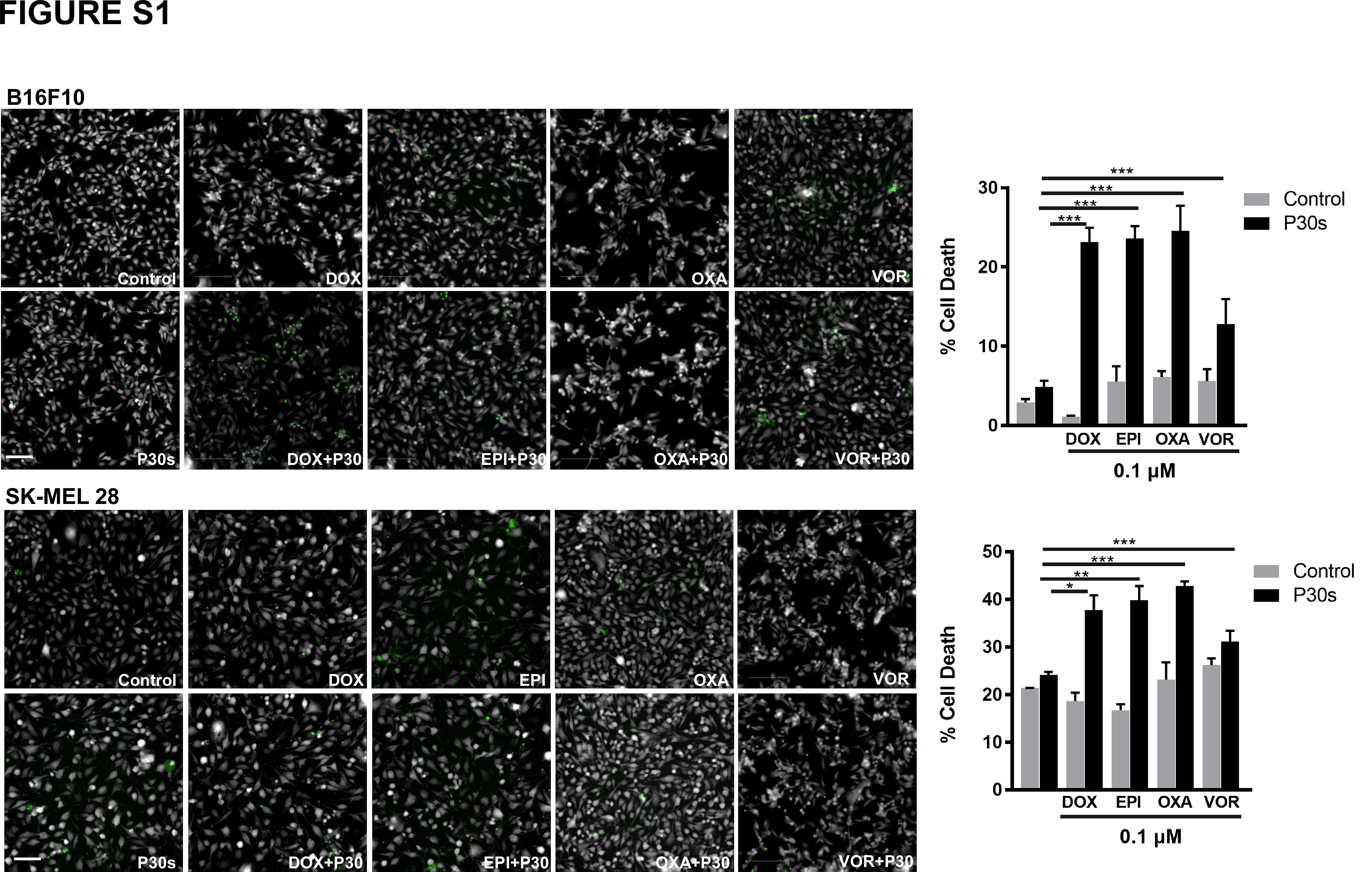

Supplement: Supplementary file 1 — Figure S1 [file 41419_2018_1221_MOESM1_ESM.jpg]

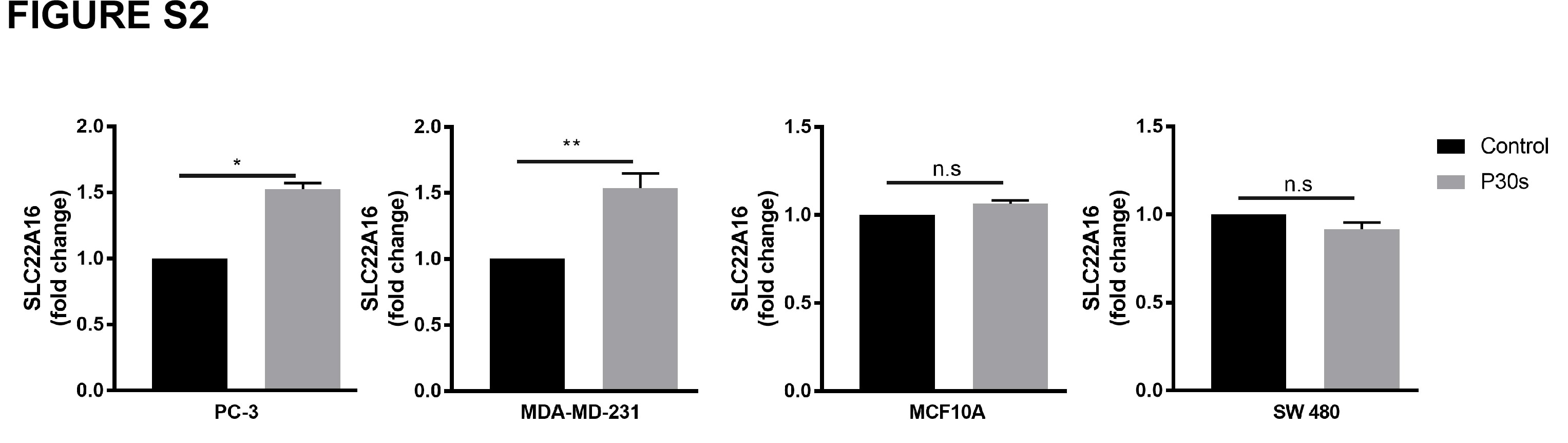

Supplement: Supplementary file 2 — Figure S2 [file 41419_2018_1221_MOESM2_ESM.jpg]

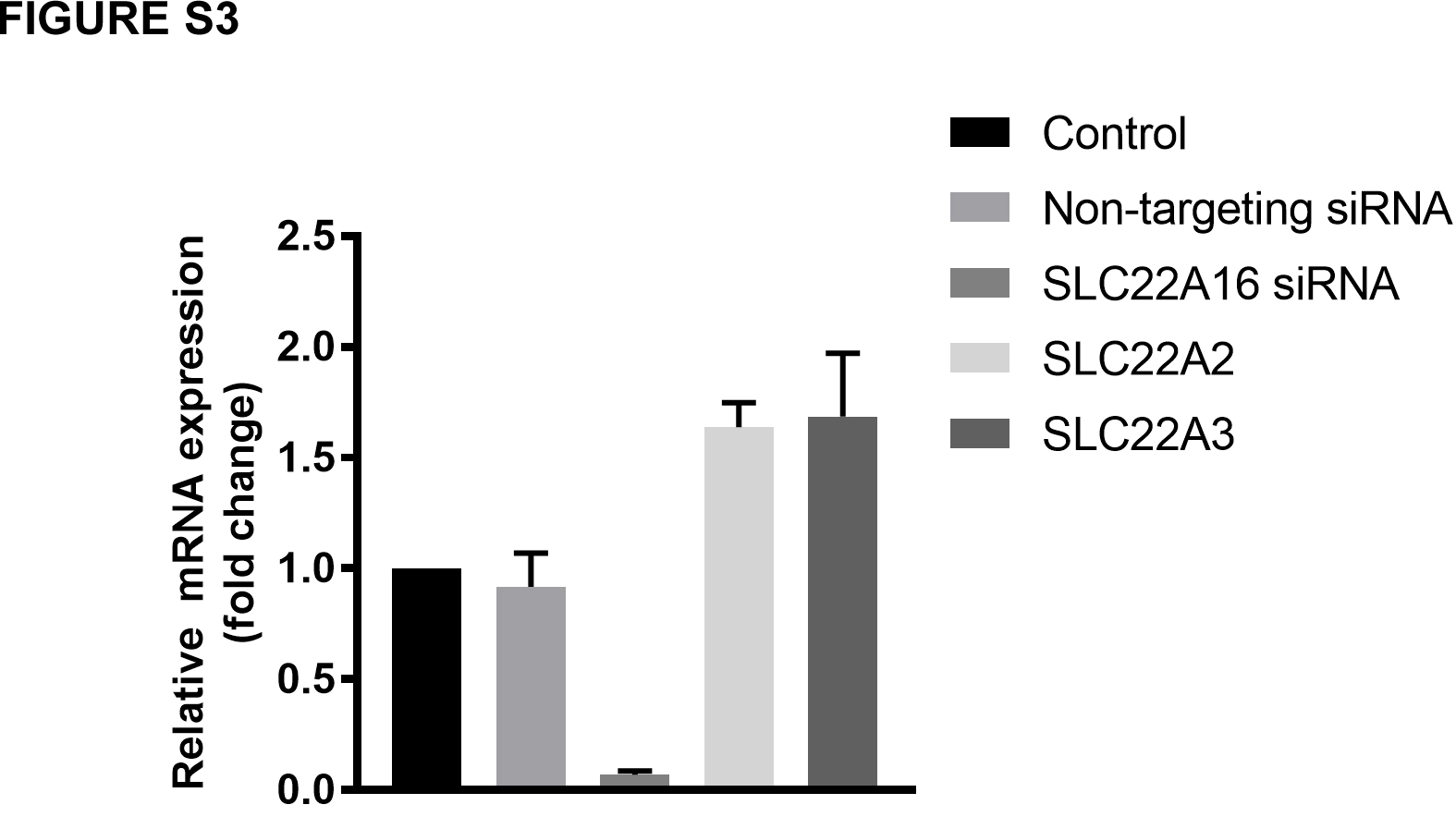

Supplement: Supplementary file 3 — Figure S3 [file 41419_2018_1221_MOESM3_ESM.jpg]
