# Supplementary material for: Combination of chemotherapy and physical plasma elicits melanoma cell death via upregulation of SLC22A16
Source: Cell Death Dis. 2018 Dec 5;9(12):1179. doi: 10.1038/s41419-018-1221-6 (PMC6281583; doi:10.1038/s41419-018-1221-6)
Supplement: Supplementary file 7 — Supplementary figure legends [file 41419_2018_1221_MOESM7_ESM.docx]

**Figure S1.** ***Sytox green viability staining*** ***in drug-sensitized melanoma cells 6 hours following plasma treatment.***

**Figure S2.** ***Expression of SLC22A16 in multiple tumor cell lines following 30s plasma treatment.***

**Figure S3*.*** ***Dosage compensation*** ***of SLC22A2 and SLC22A3 in SK-MEL 28*** ***cells following SLC22A16 esiRNA knockdown.***
